# 3D reconstruction of shoulder muscles in hominoid primates: Correlating scapular attachment areas with muscle volume

**DOI:** 10.1111/joa.14199

**Published:** 2025-01-04

**Authors:** Julia van Beesel, Stephanie Melillo, Evie Vereecke

**Affiliations:** ^1^ Department of Development and Regeneration KU Leuven Leuven Belgium; ^2^ Biomechanical Engineering TU Delft Delft The Netherlands; ^3^ Department of Human Origins Max Planck Institute for Evolutionary Anthropology Leipzig Germany; ^4^ Department of Applied Forensic Sciences Mercyhurst University Erie Pennsylvania USA

**Keywords:** muscle origin area, muscle volume reconstruction, rotator cuff, surface scanning

## Abstract

Digital muscle reconstructions have gained attraction in recent years, serving as powerful tools in both educational and research contexts. These reconstructions can be derived from various 2D and 3D data sources, enabling detailed anatomical analyses. In this study, we evaluate the efficacy of surface scans in accurately reconstructing the volumes of the rotator cuff and teres major muscles across a diverse sample of hominoids. Additionally, we investigate whether muscle origin area, as a dissection‐based observation, can reliably predict muscle volume. Our findings reveal that surface scans provide sufficient coverage to accurately reproduce the *in situ* volumes of the rotator cuff muscles. However, the volume of the teres major was estimated less reliably, suggesting that muscles with less distinct skeletal boundaries may present challenges for accurate reconstruction. Future studies will explore whether such muscles can be reconstructed with greater precision. Furthermore, we identify a significant correlation between the origin area and muscle volume for the supraspinatus, infraspinatus, and subscapularis muscles. These results suggest that muscle origin area can serve as a reliable predictor of muscle volume, offering a skeletal indicator for estimating muscle size in both extant and extinct hominoids. These insights are particularly valuable for paleontological reconstructions, where direct soft tissue evidence is often lacking. By establishing a relationship between skeletal traits and muscle volume, our study provides a framework for evaluating the accuracy of soft tissue reconstructions in hominoid species. This approach not only enhances our understanding of hominoid anatomy but also offers new avenues for exploring the functional morphology of extinct taxa.

## Graphical abstract

Digital muscle reconstructions are opening up a whole new world in understanding anatomy! In this study, we are thrilled to show that surface scans can capture rotator cuff volumes across a range of hominoids, while identifying challenges with less well‐defined muscles like the teres major. We have discovered an important link between skeletal origins and muscle size, offering a new way to estimate muscle size even in extinct species.

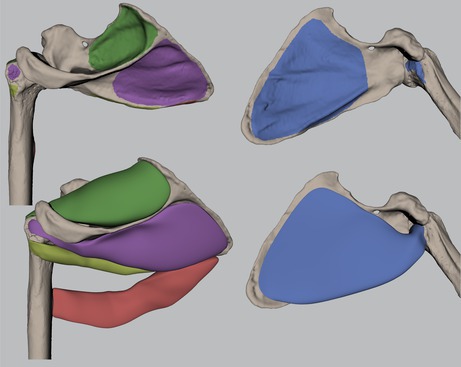

## 1 INTRODUCTION

### 1.1 Importance of muscle volume reconstructions

Digital representations and analyses of the musculoskeletal system have gained significant popularity in recent years for educational, visual, and research purposes. The pioneering effort by the Visible Human Project (Ackerman, 1998) laid the foundation for extensive datasets and advanced analytical methods that have since expanded substantially. These digital resources have increasingly been employed to study the gross anatomy of various species in a comparative context, often incorporating fossil specimens into the analyses (Demuth et al., 2022, 2023; Eigen & Nyakatura, 2021; Herbst et al., 2022; Titmus et al., 2023; Wiseman, 2023).

A central component of these *in silico* studies involves the reconstruction of three‐dimensional (3D) geometries of muscles and bones. By assembling individual musculoskeletal elements digitally, researchers can create comprehensive models that accurately represent specific body regions or entire organisms *in silico*. Reconstructed muscle geometries serve critical functions in such models, including the estimation of muscle volumes (Eigen & Nyakatura, 2021; Wiseman, 2023), which is essential for assessing the force‐producing capabilities of muscles, particularly in extinct species. Additionally, these geometries facilitate the determination of muscle lines of action (Demuth et al., 2022; Modenese & Kohout, 2020), a crucial aspect for developing musculoskeletal models used in biomechanical analyses. Through the evaluation of muscle lines of action, important biomechanical parameters such as muscle moment arms can be estimated, providing insights into the functional capacities of musculoskeletal structures. Consequently, the determination of muscle lines of action for use in musculoskeletal models has become an invaluable tool in paleontological research across a variety of extinct taxa (Demuth et al., 2023; Hutchinson et al., 2005; Wiseman et al., 2024), effectively contributing to the reconstruction of locomotor behaviour and the functional anatomy of fossil organisms.

In extant species, 3D muscle geometries are typically reconstructed from diverse imaging methods, including magnetic resonance imaging (MRI), contrast‐enhanced computed tomography (CT) scans, cross‐sectional photographs, and photogrammetry. Notably, Demuth et al. (2022) employed both cross‐sectional images and surface scanning techniques to develop 3D reconstructions of muscle geometries and volumes, marking a novel application of surface scanning in this context. Similar to photogrammetry (Titmus et al., 2023), surface scanning offers the advantage of being able to directly integrate with dissection procedures, allowing for simultaneous collection of various data types, such as joint mobility measurements and muscle fiber parameters (e.g., fiber length and pennation angle), which are also critical for constructing accurate musculoskeletal models. This kind of specimen‐specific approach to musculoskeletal modelling offers the advantage of being based on a single individual and, therefore, avoids the confounding issues involved with combining empirical data with different biological profiles. While Demuth et al. (2022) assessed the accuracy of volumetric reconstructions by comparing them to experimentally measured muscle volumes, they only did so on one gorilla specimen, precluding statistical analysis.

The first aim of the present study is to evaluate whether muscle volumes can be estimated with sufficient accuracy from 3D digital muscle reconstructions based on surface scans. Our null hypothesis (H_0__1) posits that there is no significant difference between reconstructed muscle volumes and experimentally measured volumes, indicating that surface scan‐based reconstructions are accurate. Conversely, the alternative hypothesis (H_1__1) suggests a systematic bias or significant difference between the two measurement techniques, implying that surface scanning may not provide sufficiently accurate muscle volume estimations. We will test this using a sample of nine specimens from eight different species across the superfamiliy Hominoidea, focusing on selected shoulder muscles, namely the rotator cuff and teres major muscles.

### 1.2 Role of the rotator cuff in the Hominoidea

The rotator cuff comprises the supraspinatus, infraspinatus, teres minor, and subscapularis muscles and has a key stabilizing function during the diverse locomotor activities of hominoids (Larson & Stern, 1986, 1987). Especially the supraspinatus muscle is thought to be crucial in apes, not only for stabilizing the glenohumeral joint but also for assisting the deltoid muscle during arm elevation (Inman et al., 1944; Larson, 1993). In a recent biomechanical analysis of the rotator cuff, we showed that the supraspinatus in gorillas has greater force‐producing capabilities and larger moment arms for arm‐raising compared to humans, supporting the idea that the supraspinatus assists the deltoid in this movement (van Beesel et al., 2021). Both muscle volume (which relates to force production) and muscle line of action (linked to muscle moment arm) are important biomechanical parameters that can be estimated in extant and extinct species through digital reconstructions of these muscles.

Digital reconstructions of muscles in extinct species are typically developed using the extant phylogenetic bracket approach (Witmer, 1995). This method involves studying the anatomy of extant species closely related to the fossil specimen and using that knowledge to infer the geometric properties of soft tissue of the extinct species. However, this approach presents two main challenges when studying hominoids. Firstly, detailed anatomical descriptions or digital reconstructions of closely related extant species must be available, which is challenging in the case of non‐human apes (hereafter referred to as apes) due to limited observations and restricted access to ape cadavers. Apart from the study by Demuth et al. (2022), digital reconstructions of ape rotator cuff muscles are scarce. Secondly, using the extant phylogenetic bracket to develop muscle reconstructions introduces potential bias, as the accuracy of these reconstructions cannot be validated due to the lack of fossil evidence for soft tissue. Therefore, it is crucial to have a method to assess the reliability of these reconstructions in extant primates, such as evaluating whether the reconstructed muscle volumes are plausible. Any skeletal indicators that can provide insights into muscle volume would be invaluable.

A potential skeletal indicator for the volume of rotator cuff muscles are the three scapular fossae. It has long been hypothesized that the dimensions of the scapular fossae are related to the volumes of the supraspinatus, infraspinatus, and subscapularis muscles, and vice versa (Ashton & Oxnard, 1963, 1964; Larson, 1993; Roberts, 1974; Young, 2008). However, within hominoids, this connection has been questioned by recent studies, which found that the relationship between fossa dimensions and muscle masses (used as a proxy for muscle volume) is not as strong as previously believed (Bello‐Hellegouarch et al., 2013; Larson & Stern, 2013). These studies observed conflicting results when comparing relative scapular fossa sizes and muscle masses across different species. Importantly, most of these studies did not analyse the actual 3D area of muscle origin but relied on linear measurements of the fossae and did not collect both muscle origin areas and volumes from the same individuals. To date, the only study that did use an integrated dataset (Bello‐Hellegouarch et al., 2013) did not perform a correlation analysis between these traits but rather compared indices of each trait between different hominoid species.

To address these shortcomings, the current study uses an integrated dataset of nine hominoid specimens to investigate the correlation between muscle origin area and volume of the rotator cuff. Specifically, we seek to determine whether muscle origin area and muscle volume are correlated and test whether muscle volume can be accurately predicted based on the size of the origin area on the scapula. Our null hypothesis (H_0__2) is that there is no correlation between the muscle origin area and the experimentally measured volume of the rotator cuff. Conversely, our alternative hypothesis (H_1__2) posits that there is a significant correlation between muscle origin area and volume. By exploring this relationship, we aim to contribute to the understanding of the link between skeletal (origin area) and muscle (volume) of the rotator cuff muscles in hominoids, potentially offering implications for both extant and extinct species.

Additionally, we have included the teres major muscle in both our surface scan muscle geometry reconstructions and origin area‐to‐volume correlation analyses. The teres major is less constrained by bony structures compared to the rotator cuff muscles, and previous research suggested that muscles with fewer skeletal constraints are at greater risk of being inaccurately reconstructed (Demuth et al., 2022). Therefore, it seemed important to include at least one muscle of this type in our analysis. Moreover, in recent research (van Beesel et al., 2022) we found that, in gorillas, the teres major muscle has force‐producing capacities and moment arms better suited for arm adduction compared to humans, which may be critical for climbing in gorillas and potentially also in other apes. Consequently, precise digital reconstructions of the teres major to analyse muscle volume (as a proxy for force) and muscle line of action (as a proxy for moment arm) are essential to compare the functional capacity of shoulder muscles across hominoids.

## 2 MATERIALS AND METHODS

### 2.1 Specimen details

The details of the nine hominoid specimens used for this study are provided in Table 1. The dissection of the female gorilla specimen has been extensively described in two previous studies (van Beesel et al., 2021, 2022). Data from this specimen were also used in another study Demuth et al. (2022). For all other specimens, the data were newly collected and not previously described or analysed. It is important to note that a full dataset was collected for all specimens except for three (ID numbers 129, 130, and 131, see Table 1). For these three specimens, only muscle attachment size and volume were collected to expand the sample size for the area‐to‐volume correlation analysis. To allow comparison with human data, we collected data from a human body donor using the same protocol. The human body was obtained with written consent from the donor for use in research following the regulations of the human body donation program of the University of Ghent. All specimens died under natural circumstances and did not display signs of musculoskeletal pathologies. They were frozen shortly after death and stored at −20°C until imaging and experiments took place. The specimens were thawed at room temperature for one to 3 days prior to the dissections.

**TABLE 1:** Specimen data.

Specimen ID	Species	Sex	Age at death (years)	Collection
123	*Hylobates lar*	Male	44.7	Réserve Africaine de Sigean, FRA
122	*Symphalangus syndactylus*	Male	26.2	DierenPark Amersfoort, NL
3	*Pongo pygmaeus*	Male	36.0	Erie Zoo, PA, US
129	*Pongo abelii*	Female	23.0	Zoo Planckendael, Mechelen, BE
2	*Gorilla gorilla*	Female	48.8	Erie Zoo, PA, US
131	*Gorilla gorilla*	Male	27.1	Blijdorp, Rotterdam, NL
1	*Pan paniscus*	Male	8.5	Twycross Zoo, GBR
130	*Pan troglodytes*	Female	40.5	Blijdorp, Rotterdam, NL
36	*Homo sapiens*	Male	81.0	UZ Gent, BE

### 2.2 Imaging and dissection protocol

Three‐dimensional (3D) digital representations of musculoskeletal structures were acquired using two imaging techniques at various stages of the data collection process, namely CT scanning and surface scanning. Full body CT scans were taken prior to performing dissections for all specimens, except for the bonobo specimen (*Pan paniscus*), whose individual bones were scanned only after dissection. The CT scanning was done at local hospitals or veterinary facilities using clinical CT scanners. While imaging settings varied, we ensured that scan parameters were defined and measurements were accurate, making the resulting dimensions comparable across all specimens. The CT scans were used to create 3D polygonal models of the skeletal structures of the shoulder (i.e., humerus and scapula). Surface scanning was used to capture 3D muscle geometry and attachment sites on the bones during dissection. The surface scanning protocol follows van Beesel et al. (2021). After removing the skin from the specimen, muscle geometry was captured layer by layer, from superficial to deep. Teres major and minor muscles were captured first, followed by the supra‐ and infraspinatus muscles, and finally the subscapularis muscle. Prior to scanning each layer, the arm was positioned and fixed either raised or lowered based on the visibility of the muscle geometries. In addition, connective tissue and fascia were removed from the muscles and each muscle was marked along its midline with dissection pins, using a different colour for each muscle to enhance later identification. A surface scan to capture the 3D geometry of the muscles and the midline pins was conducted using an Artec Space Spider and Artec Studio 18 software. Subsequently, the muscles were excised, while their origin and insertion sites were simultaneously pinned on the bones. Another surface scan was taken to document the pinned attachment sites of each muscle. This procedure was followed for each muscle layer. Each excised muscle was weighed and the muscle‐tendon length was recorded using a digital caliper. The experimental muscle volumes are calculated by dividing the measured muscle mass by the standard muscle density for mammalian muscle tissue $1.06*10^{3}\frac{\mathrm{kg}}{m^{3}}$ (Mendez, 1960; Ward & Lieber, 2005).

### 2.3 3D muscle reconstruction

Bone polygonal models of the scapula and humerus were generated from CT scans using the threshold method in 3D Slicer (https://www.slicer.org/, see Kikinis et al. (2014)), and were manually refined as necessary. The bone segmentation labels derived from CT scans were exported as 3D bone meshes (.obj file format) from 3D Slicer. Due to the size limitations of the scanner, only a partial scan of the *Pongo pygmaeus* shoulder could be performed, covering only the proximal half of the humerus. Consequently, a 3D surface scan was performed after removing all soft tissue from the humerus to generate a full 3D bone mesh. All complete 3D bone meshes can be found in our online zenodo repository (van Beesel, Melillo & Vereecke, 2024).

**FIGURE 1:**
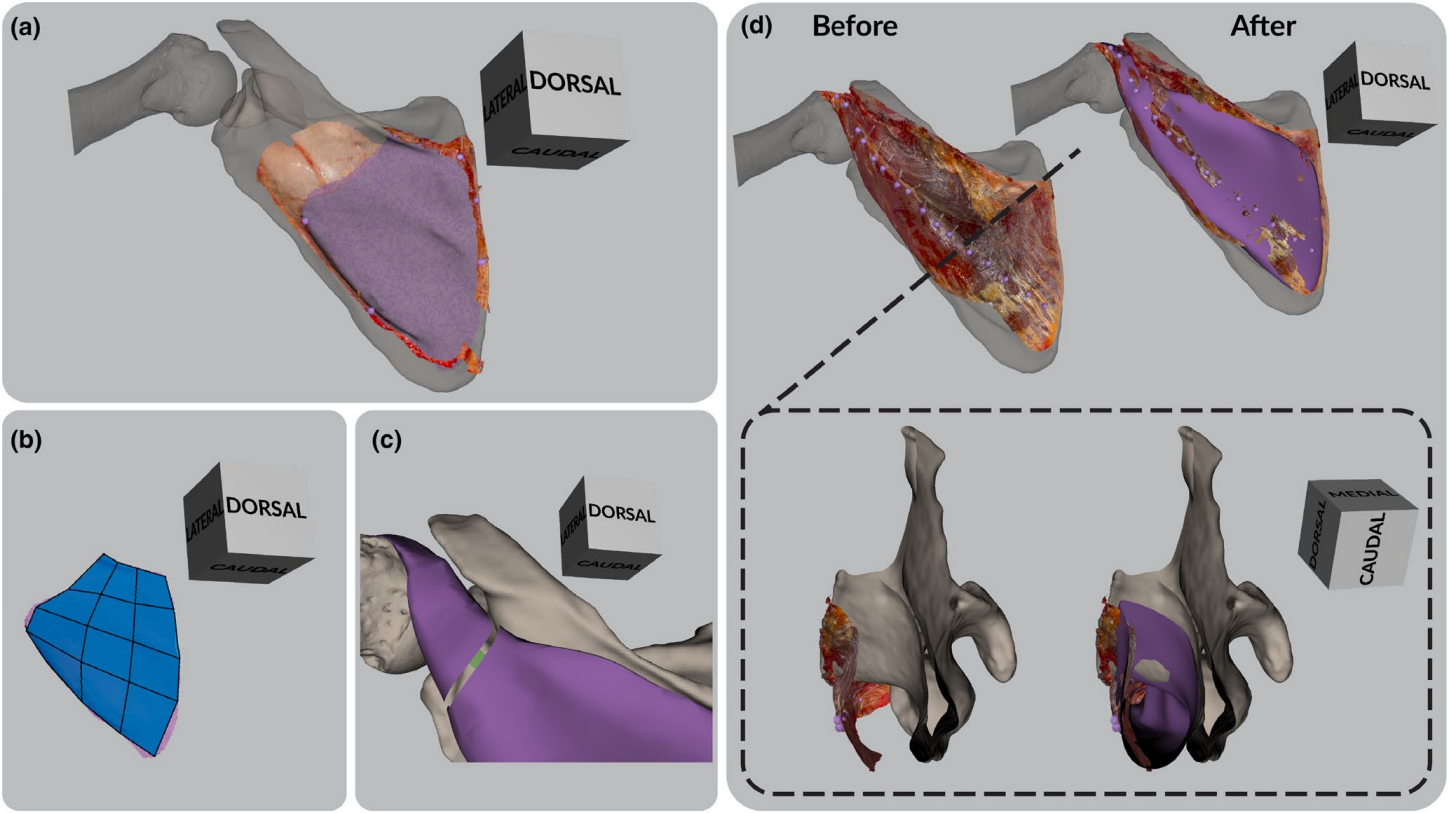
Workflow of the muscle volume reconstruction using the example of the infraspinatus muscle of the *Pongo pygmaeus* specimen. (a) The different coloured pins (here purple) visible on the surface scan mark the muscle origin area as observed during dissection, and guide the virtual 3D muscle origin area reconstruction (purple polygon). (b) Drawing faces (transparent blue) onto scapular origin area of infraspinatus muscle (purple) to initiate volume reconstruction. (c) After extruding faces from both origin and insertion site, appending both halves of the muscle volume (purple) with each other (connecting face in green). (d) Muscle volume reconstruction before and after completion. Shown here is how the muscle volume reconstruction fills in the empty space between the surface scan and the CT bones, each constraining the boundaries of the muscle volume. The left images (“before”) show the alignment of the surface scan and CT bones prior to reconstruction, while the right images (“after”) show the finalised muscle volume in purple. The top row presents a dorsal view of the surface scan, scapula, and humerus, and the bottom row shows a cross‐section through the infraspinous fossa (position indicated by a black dashed line in the top left image). In both perspectives, the surface scan and CT bones guide the muscle reconstruction by defining the available space for the 3D infraspinatus model.

The surface scans were reconstructed in Artec Studio 18 software. The scans were merged into complete polygonal models with texture embedded, which facilitated the identification of muscles and coloured pins. All surface scans were first superimposed on the scapula mesh derived from CT scan, with reference to bone‐pinned landmarks, in reverse order from deep to superficial layers. Next, the humerus mesh was aligned to the surface scans that had captured the articulated joints and muscle geometries. This usually resulted in three different humerus orientations, one for each of the three layers. The surface scans that showed the muscle attachment sites were then duplicated and the copies were aligned with the corresponding humerus orientation. Next, the attachment sites were segmented by outlining the pinned areas on the surface scan and projecting them onto the underlying CT bone mesh. As a result, the surface scans facilitated the precise transfer of the *in situ* observations regarding muscle attachment areas onto the *in silico* CT bones. The segmentation was then inverted, to end up with only the attachment area as a 3D surface (Figure 1a).

The muscle volume reconstruction was conducted in Maya 2023, adhering to the detailed iterative polygonal modelling methodology described in Demuth et al. (2022). The 3D bone polygons, muscle attachment areas, and muscle geometry surface scans (including texture) were imported into Maya and used to drive and constrain the muscle volume reconstructions. Each muscle volume was manually reconstructed using the muscle attachment sites as points of origin and extruding the meshes from there, following the bone contours and muscle geometry of the surface scans. The Quad Draw Tool was utilized to draw faces directly onto the muscle's origin area on the scapula in live mode (Figure 1b). Subsequently, the muscle was formed by extruding new faces and shaping them with the transformation tools. As the muscle volume approached the insertion site, the Quad Draw Tool was again used to draw faces on the humeral insertion area. Faces were then extruded until they connected with the previously reconstructed muscle. The gap between the two sections was closed using the Append To Polygon Tool (Figure 1c). The volumes were adjusted one final time in order to ensure the closest possible fit between the surface scan and the CT bones (Figure 1d). Then, the volume reconstructions were smoothed and triangulated in Maya and exported as .obj files.

### 2.4 Data analysis

The reconstructed muscle volumes and attachment surfaces were imported into 3D Slicer to determine muscle volume size, muscle length, and attachment area size. The surface files were converted to segmentation nodes, and the segment statistics tool was used to measure surface area (cm^2^) and surface volume (cm^3^). Markup curves were used to draw a curve on the outer surface of the reconstructed muscles, starting approximately in the center of the origin and, following the midline of the muscle, going to the center point of insertion. The markup evaluation was used to measure the length of the curve as an approximation of muscle‐tendon unit length.

The statistical tests and accompanying figures were run in custom Python script, which, together with the muscle origin and volume measurements, can be viewed online (https://juliavanbeesel.github.io/ShoulderMuscleReconstructions/intro.html) and accessed through our zenodo repository (van Beesel, Melillo & Vereecke, 2024).

To evaluate the precision of the muscle volume reconstructions, we compared the reconstructed muscle volumes—derived from polygons—with the experimental volumes. Given the significant size variation among the specimens studied and the lack of body mass data for many, we applied a logarithmic transformation to all data prior to performing the statistical tests. Without this adjustment, larger specimens would disproportionately influence the tests, leaving smaller specimens underrepresented. Furthermore, the transformation has the advantage that our results can be used for other studies where no body mass information is available either. The precision was evaluated by conducting a paired *t*‐test and by calculating the mean standard error, root mean squared error, and coefficient of determination using custom written Python code. We used a *p*‐threshold of 0.05 to determine the statistical significance of our observations. The agreement between the reconstructed and experimentally measured volumes is evaluated using a Bland–Altman plot.

To validate the use of muscle origin area as a predictor for muscle volume, we performed a series of analyses comparing experimentally measured muscle volumes with muscle origin areas—as identified during dissections—of the rotator cuff and teres major muscles in hominoids. First, we applied the Shapiro–Wilk test to assess the normality of each dataset (log‐transformed muscle origin area and experimentally measured volume). The outcomes of the Shapiro–Wilk test are available in the supplementary information. Upon confirming the normal distribution of the data, we calculated the Pearson correlation coefficient for the paired dataset of all rotator cuff and teres major muscles across all species. From this analysis, we derived the equation for a linear regression of the paired muscle origin areas and volumes.

During initial data exploration using scatter plots, we observed that the supraspinatus, infraspinatus, and subscapularis muscles displayed a similar linear relationship between origin area and volume, distinct from that of the teres major and teres minor muscles. Based on this observation, we decided to perform separate correlation analyses for these muscles, akin to the work by Cuff et al. (2022). This methodological decision is explained in further detail in the Results section.

During the preparation of this work we used ChatGPT (version GPT‐4) for evaluating and improving the performance of the Python code and for the improvement of readability and language.

## 3 RESULTS

### 3.1 Muscle origin area and volume reconstructions

**FIGURE 2:**
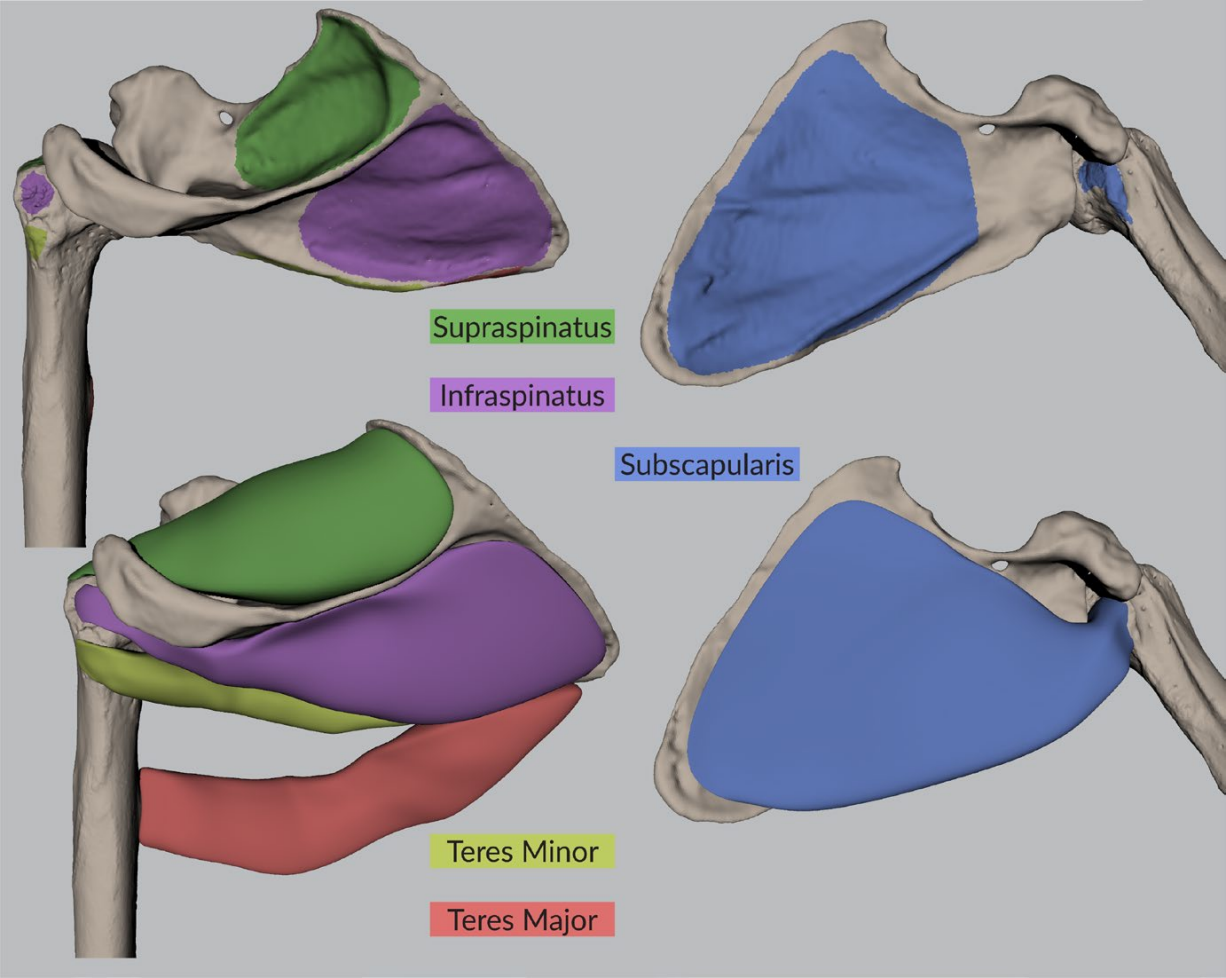
Muscle attachment sites and reconstructed rotator cuff and teres major muscles of the *Pongo pygmaeus* specimen (see also Figure 1).

The experimental and reconstructed measurements of muscle origin area, length, and volume are given in Table 2. The table also shows the percentage difference between the reconstructed and the experimental volumes and lengths. Negative values indicate an underestimation, positive values an overestimation. The absolute differences in lengths are minimum 1.0% and maximum 26.9% and the absolute differences in volumes are minimum 0.3% and maximum 54.5%. For both length and volume measurements, the errors are highest for the teres major (first) and minor (second) muscles. Figure 2 illustrates the muscle attachment sites and muscle volume reconstructions for the *Pongo pygmaeus* specimen.

**TABLE 2:** Measurements of origin area, length, and volume of both reconstructed and experimental muscles.

Muscle name	ID	Genus	Origin area (cm^2^)	L rec (cm)	L exp. (cm)	L diff (%)	V rec (cm^3^)	V exp. (cm^3^)	V diff (%)
Supraspinatus	123	*Hylobates*	7.5	7.2	8.0	−9.9	7.5	7.7	−3.3
	122	*Symphalangus*	9.4	8.9	9.8	−9.7	12.5	14.7	−15.0
	3	*Pongo*	41.5	16.5	15.2	8.4	129.0	145.0	−11.0
	129	*Pongo*	21.5	—	10.8	—	—	43.4	—
	2	*Gorilla*	47.7	19.4	19.2	1.1	94.1	79.2	18.8
	131	*Gorilla*	146.2	—	19.4	—	—	376.4	—
	1	*Pan*	19.4	10.2	10.6	−4.4	38.2	40.6	−5.8
	130	*Pan*	31.2	—	13.1	—	—	36.5	—
	36	*Homo*	36.7	17.3	15.1	14.6	40.8	36.5	11.7
Infraspinatus	123	*Hylobates*	12.3	10.5	10.6	−1.0	11.2	11.2	0.3
	122	*Symphalangus*	18.5	12.5	14.6	−14.4	22.2	25.1	−11.5
	3	*Pongo*	87.3	23.6	22.9	3.2	187.7	208.6	−10.0
	129	*Pongo*	57.0	—	18.8	—	—	93.5	—
	2	*Gorilla*	79.0	26.1	26.6	−1.8	123.9	98.9	25.3
	131	*Gorilla*	187.7	—	31.0	—	—	575.3	—
	1	*Pan*	30.7	16.4	18.1	−9.7	71.2	84.9	−16.1
	130	*Pan*	51.5	—	19.1	—	—	77.6	—
	36	*Homo*	65.7	17.8	19.5	−9.0	95.9	77.6	23.7
Subscapularis	123	*Hylobates*	16.5	9.2	7.9	17.1	11.0	13.1	−16.1
	122	*Symphalangus*	36.0	13.3	13.5	−2.1	49.0	45.8	6.9
	3	*Pongo*	127.2	19.3	21.5	−9.9	241.1	327.1	−26.3
	129	*Pongo*	78.8	—	18.7	—	—	156.9	—
	2	*Gorilla*	132.7	25.4	24.1	5.2	253.6	225.6	12.4
	131	*Gorilla*	339.3	—	29.2	—	—	973.4	—
	1	*Pan*	60.0	18.2	17.2	5.4	154.0	133.0	15.8
	130	*Pan*	87.1	—	14.4	—	—	109.2	—
	36	*Homo*	104.5	18.5	18.8	−1.4	130.1	109.2	19.1
Teres Minor	123	*Hylobates*	1.3	6.0	5.4	11.4	2.3	2.4	−3.1
	122	*Symphalangus*	1.4	7.1	8.8	−19.6	8.0	7.1	13.0
	3	*Pongo*	4.1	15.4	17.5	−12.0	41.5	86.7	−52.1
	129	*Pongo*	3.0	—	11.4	—	—	29.2	—
	2	*Gorilla*	7.2	12.8	15.9	−19.4	19.2	23.7	−19.2
	131	*Gorilla*	9.9	—	20.5	—	—	105.1	—
	1	*Pan*	2.2	10.8	14.1	−22.9	14.8	17.0	−12.6
	130	*Pan*	3.6	—	12.3	—	—	17.2	—
	36	*Homo*	9.1	12.7	17.4	−26.9	17.7	17.2	2.4
Teres Major	123	*Hylobates*	2.7	10.5	9.7	8.6	21.9	14.2	54.5
	122	*Symphalangus*	2.7	12.3	14.1	−12.2	37.0	24.6	50.1
	3	*Pongo*	6.0	21.1	28.1	−25.0	105.1	184.8	−43.1
	129	*Pongo*	5.8	—	21.7	—	—	115.0	—
	2	*Gorilla*	17.6	26.5	23.6	12.2	115.2	97.0	18.8
	131	*Gorilla*	—	—	34.1	—	—	768.0	—
	1	*Pan*	3.2	14.4	18.0	−20.0	78.2	127.4	−38.6
	130	*Pan*	3.8	—	19.8	—	—	64.4	—
	36	*Homo*	9.1	25.2	21.8	16.0	31.0	64.4	−51.8

*Note*: The table shows reconstructed (rec) and experimental (exp) values for muscle lengths (*L*) and volumes (*V*). Differences (diff) between experimental and reconstructed values are given in percentage (%).

### 3.2 Accuracy of muscle volume and length reconstruction

**FIGURE 3:**
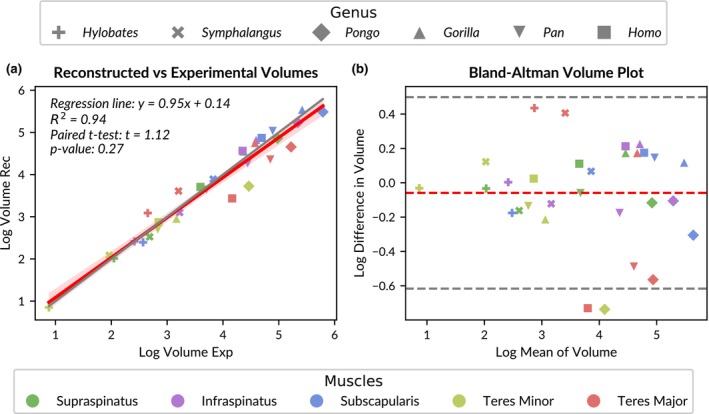
Accuracy of muscle volume reconstructions. (a) Linear regression of reconstructed (rec) muscle volumes compared to experimentally (exp) measured ones. The red line illustrates the optimal linear regression fit, surrounded by a red shaded area representing the 95% confidence interval of that regression. The grey line represents the identity line. (b) Bland–Altman plot displaying the differences between log‐transformed experimental and reconstructed muscle volumes. The mean difference between the two measurements is shown by a red dashed line, while the grey dashed lines indicate the limits of agreement, which are determined as the mean difference ±1.96 times the standard deviation of the differences.

The paired *t*‐test suggests that the two different volume measurement methods yield similar means and variations in log‐transformed volumes, with no evidence of systematic error (*t*‐statistic: 1.12, *p*‐value: 0.27). The mean absolute error (MAE: 0.22) and root mean squared error (RMSE: 0.29) on the log‐transformed scale are relatively low. The coefficient of determination is *R* ^2^: 0.940 (see Figure 3a). We further assessed whether the regression line significantly deviates from the identity line (slope = 1, intercept = 0). The analysis showed that the slope does not significantly differ from one (*t*: −1.17, *p*: 0.25), indicating an absence of proportional bias, and the intercept does not significantly differ from zero (*t*: 21.435, *p*: 0.44), indicating no constant bias. Given the high *p*‐values, we found no evidence to reject the null hypothesis (H_0__1), suggesting no significant difference between the two measurement methods.

This is further confirmed by the Bland–Altman plot (Figure 3b). The mean difference between the two muscle volume measurements is close to zero (−0.06). The only two outliers (beyond the limits of agreement) are a reconstruction of a teres minor (orangutan) and teres major (human) muscle volume, which significantly underestimate the experimental muscle volumes. Notably, teres major volume reconstructions generally deviate the most from experimental volumes, indicating that reconstructions of this muscle type have the highest uncertainty.

The muscle length measurements obtained from the reconstructed volumes are significantly different from the experimental measurements (*p*‐value: 0.04). Consequently, the reconstructed muscle lengths do not accurately replicate the lengths measured experimentally. Detailed statistical test results and a Bland–Altman plot illustrating the findings are provided in the Data S1.

### 3.3 Correlation analysis

**FIGURE 4:**
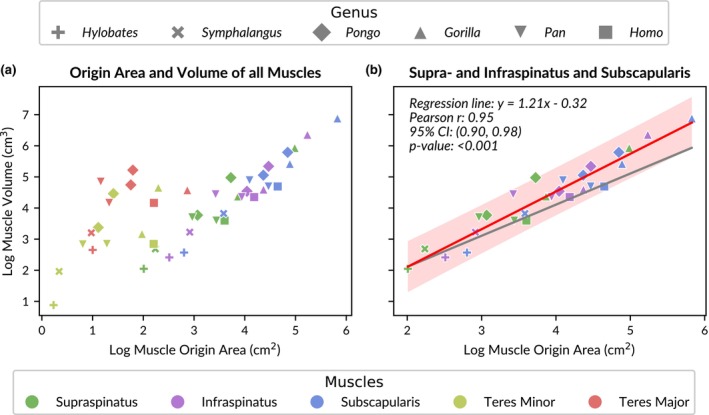
Analysis of the correlation between log‐transformed muscle volume and origin area. (a) Scatter plot illustrating volumes versus origin areas for all muscles. (b) Pearson correlation of muscle origin areas and volumes of the supraspinatus, infraspinatus, and subscapularis muscles. The red line shows the best‐fit linear regression between muscle origin area and volume. The red shaded area around the red line indicates the prediction intervals. The grey line represents the prediction assuming isometric scaling with a slope of one.

The scatter plots suggest that there is a linear association between the log‐transformed origin area [cm^2^] and muscle volume [cm^3^] of the supraspinatus, infraspinatus, and subscapularis muscles (Figure 4a). This relationship was further explored using a correlation analysis. As the data points for the teres major and teres minor muscles do not adhere to this linear trend, the correlation between origin area and volume of these muscles was analysed separately (see the Data S1 for details).

The correlation between the log‐transformed origin area and the experimentally measured muscle volume for the supraspinatus, infraspinatus, and subscapularis muscles was evaluated using Pearson's correlation coefficient (Figure 4b). The analysis showed a strong, positive correlation between the two variables, *r* = 0.95 (95% Confidence Interval: 0.90, 0.98), which was statistically significant, *p* < 0.001 (df = 25; *n* = 27). This supports our alternative hypothesis (H_1__2).

These findings indicate that muscle origin area is strongly correlated to muscle volume for these three muscles across all included specimens in this portion of the study. As the slope of the regression line equation is greater than one (*y* = 1.21*x*–0.32), it appears that this relationship is hyperallometric (positive allometry; compare to black line in Figure 4b).

The Pearson correlation results for origin area and muscle volume of teres major and minor (see the Data S1) reveal a moderate but significant positive correlation (*r* = 0.66, *p* < 0.01). Nevertheless, the wide range of the confidence interval limits for the correlation coefficient (CI: 0.26, 0.86) indicate substantial uncertainty, likely in part due to the large variability in the data and the relatively small sample size (df = 15).

## 4 DISCUSSION

The results of this study show that (1) muscle volumes can be accurately estimated from volumetric reconstructions using surface scans of carefully dissected muscle layers, supporting the first null hypothesis (H_0__1), and (2) the origin area on the scapula of the supraspinatus, infraspinatus, and subscapularis muscles can be used to predict muscle volume with reasonable accuracy, following from the second alternative hypothesis (H_1__2).

This relationship enables us to derive a linear regression equation between logarithmic transformed muscle volume and origin area, which can serve as a predictive tool in future studies. Such a tool is particularly valuable for estimating muscle volumes or validating muscle volume reconstructions when direct physical measurements are not feasible. Given that this relationship was observed across multiple hominoid species, it holds significant potential for paleoanthropological research, especially in the reconstruction of muscle volumes in extinct hominoid species, which is becoming more popular (Wiseman, 2023). Since fossils do not preserve any direct muscle soft tissue information, skeletal evidence becomes crucial in inferring soft tissue characteristics. Therefore, our results, in combination with existing methods such as the extant phylogenetic bracket (Witmer, 1995), can guide muscle volume reconstructions in extinct species.

### 4.1 Reconstructing muscle volume and muscle length

The outcomes of the statistical tests demonstrate that there is no biased error between the two volume measurement methods, and that the muscle volume reconstructions align closely with the experimental volumes. Although surface scans generally do not capture the entire muscle volume, they nonetheless provide sufficient data for accurate muscle volume reconstructions. Therefore, our analysis, using a wider array of muscles and species, strengthens previous assumptions that muscle volumes can be accurately reconstructed from 3D surface scans (Demuth et al., 2022). Consequently, this suggests that muscle lines of action can be accurately inferred from surface scans and reconstructed muscle volumes (van Beesel et al., 2021, 2022). This conclusion is likely to hold true even when muscles are reconstructed from photogrammetry‐based applications as an alternative to surface scanning. Nevertheless, scanning multiple layers might be necessary for both methods, and a precise digital representation of the underlying skeleton remains crucial.

Our findings are consistent with the previous research of Demuth et al. (2022), which highlighted that the volume of muscles closely constrained by bony structures can be reconstructed with greater reliability than muscles that are less constrained. In the current study, the teres major muscle, which is surrounded by soft tissues rather than bony constraints, showed the highest level of uncertainty in volume estimation derived from surface scans. These scans, obtained primarily from the dorsal and lateral perspectives, lacked depth information from the ventral and medial sides, contributing to this uncertainty. To improve the precision of the teres major reconstructions, and hence muscle volume estimations, integrating adjacent muscles—such as the latissimus dorsi, biceps brachii, and triceps brachii—into the reconstructions could be advantageous, in line with recommendations from previous studies (Wiseman, 2023).

The volumes of the supraspinatus and infraspinatus muscles of the gorilla investigated in this study were also reconstructed in the work of Demuth et al. (2022). In that previous research, Demuth et al., who developed the workflow and have substantial expertise in virtual reconstructions and modelling, achieved reconstructed muscle volumes that closely matched the experimental volumes (supraspinatus volume: 79.44 cm^3^, difference: 0.30%; infraspinatus volume: 99.73 cm^3^, difference: 0.84%). In comparison, the muscle volumes obtained by the authors of the current study (supraspinatus volume: 94.1 cm^3^, difference: 18.8%; infraspinatus volume: 123.9 cm^3^, difference: 25.3%, see Table 2) show larger discrepancies, which may reflect differences in experience, as the authors were new to the iterative polygonal modelling approach. Although these reconstructions were performed “blind” (without prior knowledge of the measured volumes), the method produced overall reliable results, demonstrating that surface scans can enable accurate muscle volume estimation. However, this also suggests that the accuracy of such reconstructions is influenced by the expertise of the modeller and the choices made during the reconstruction process. Researchers interested in performing similar analyses can use our dataset to test the reliability of their own reconstructions. The complete dataset, comprising all surface scans of muscle origins and geometries, for two specimens (*Pongo* and *Symphalangus*), has been made available in our Zenodo repository for use as a training dataset by those new to the method (van Beesel, Melillo & Vereecke, 2024).

The findings of our study have broader implications as there are many methods next to surface scanning that allow 3D muscle volume reconstruction and that could be used for muscle volume estimations when dissection of the specimen is not possible. The choice of data acquisition method—whether DiceCT, MRI, cross‐sections, photogrammetry, or surface scanning—is dependent on the required resolution, the intended application, and the available time and resources. Techniques like DiceCT, cross‐sections, and MRI allow to capture 3D shapes of all muscles at once with the specimen in a fixed position, minimising discrepancies between layers that might arise when using photogrammetry or surface scanning. However, these techniques can be prohibitively expensive, especially when used for large‐bodied specimens or when applied to a substantial number of samples. In contrast, photogrammetry and surface scanning are less expensive and can be combined with other anatomical data collection procedures, such as measuring joint mobility or muscle fiber length. The advantage of surface‐scanning over photogrammetry is that it allows for real‐time evaluation during dissection, ensuring complete coverage of the sample area, yet this also comes at a higher cost. Finally, surface scanning, photogrammetry, and cross‐sections allow the capture of texture, which is valuable for anatomical illustrations.

Contrary to muscle volume reconstruction, estimating muscle lengths from reconstructed muscle volumes proved more challenging. Several factors may contribute to the discrepancy between estimated and measured muscle lengths. Firstly, while muscle length was measured along the midline from the center of one attachment to the other (both experimentally and on the reconstructed volumes), variability in sampling positions due to the lack of clear anatomical landmarks likely introduced significant error. Secondly, experimental measurements were taken with muscles laid flat but not stretched, whereas surface scans were performed with the arm either raised or lowered to optimize muscle volume capture, potentially resulting in stretched or compressed muscles and distorted length measurements. Lastly, during dissection, muscle length was measured in a straight line using calipers, whereas digital measurements accounted for the 3D curvature of the muscle surfaces. These methodological differences likely contributed to the observed differences between experimentally measured muscle lengths and those estimated from 3D reconstructions.

### 4.2 Correlation between muscle origin area and muscle volume

The significant positive correlation between muscle origin area and volume for the supraspinatus, infraspinatus, and subscapularis muscles has important implications for understanding the functional anatomy and evolution of hominoids. This finding suggests that muscle volume can be estimated with high accuracy from the attachment area on the scapula. Given the large amount of intact scapulae from extant hominoid species in museum collections, this opens up a wide range of research opportunities. For extinct species, this correlation could provide valuable insights by enabling muscle volume estimations from fossilized remains. However, it is crucial that this correlation be validated for extinct and other primate taxa, and it relies on the availability of intact scapulae to fully expand its applicability.

Although both age and sex are known to influence muscle volumes, our analysis reveals a statistically significant relationship between muscle origin area and volume across the hominoid sample. However, further research with larger sample sizes and specific tests is needed to determine the consistency of this relationship across different age groups and sexes. While it is understood that muscle volume can fluctuate throughout an individual's lifetime and in response to activity levels, and previous studies have shown that muscle attachment areas are not affected in a similar manner (Rabey et al., 2015), these fluctuations in volume do not appear to significantly disrupt the correlation between muscle origin area and volume observed in our study. This finding revives a previously discarded concept within hominoid research, suggesting that the development of scapular fossa areas is interconnected and linked with the associated muscle volumes (Bello‐Hellegouarch et al., 2013; Larson & Stern, 2013).

Whether this relationship between the origin area and volume of supraspinatus, infraspinatus, and subscapularis muscles extends beyond hominoids remains to be determined. Additionally, it seems plausible that other muscles, particularly those with large volumes situated directly over broad origin sites (such as the gluteal or masticatory muscles), might exhibit similar correlations between origin area and muscle volume. Future research should investigate these possibilities across different muscle groups.

In contrast, the relationship between origin area and volume was less straightforward for the teres major and minor. Both muscles originate from the axial border of the scapula, with much of their volume positioned along this border. Our dataset was too small to analyse the teres major and minor individually; however, it is possible that the teres minor muscle—which is more closely associated with the scapula—would have its own distinct correlation slope. Further investigation including a larger number of specimens is needed to look into this.

### 4.3 Comparison to previous studies

The investigation into the relationship between scapular fossae and muscle volume, and ultimately locomotor behaviour, has a long history in comparative anatomical and biological anthropological research (Ashton & Oxnard, 1963; Churchill et al., 2013; Larson & Stern, 2013; Roberts, 1974). A notable meta‐analysis by Larson and Stern (2013) compared various methods and indices used to assess the relationship between fossa size and muscle mass. They found that the 3D area measurements introduced by Bello‐Hellegouarch et al. (2013) were superior predictors of supraspinatus to infraspinatus muscle mass compared to traditional linear measurements. Additionally, they observed that excluding the teres minor from analyses improved accuracy, although this did not fully account for discrepancies in all cases.

**FIGURE 5:**
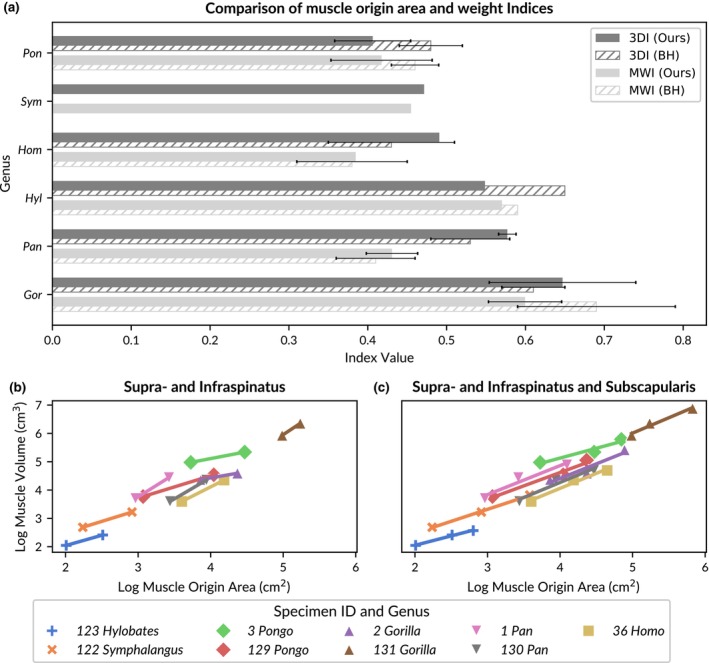
Comparison of muscle origin area and weight indices between our approach and previous research. (a) Horizontal bar plot comparing the muscle origin area index (3DI) and muscle weight index (MWI) for each genus in our dataset (labelled “Ours”) and the dataset published in table VIII of Bello‐Hellegouarch et al. (2013) (labelled “BH”). Genus names are abbreviated to the first three letters (refer to Table 1 for full names). The bars are ordered by ascending 3DI values from our dataset, with standard deviation error bars shown where available. (b) Scatter plot with individual linear regressions for each specimen, comparing log‐transformed muscle origin area and volume for the supraspinatus and infraspinatus muscles. (c) Similar to (b), but data points for the subscapularis muscle are included in the regressions.

The study by Bello‐Hellegouarch et al. (2013) remains indeed notable. While earlier research hinted at a connection between fossa area and muscle volume, their study concluded that the relationship was complex and required caution when inferring muscle size from fossa dimensions. Figure 5a compares their findings with the results of the current study. Bello‐Hellegouarch et al. (2013) measured the scapular origin areas of dorsal rotator cuff muscles and the corresponding muscle masses through dissection, similar to the approach taken in our study. They introduced two indices—one for origin area (3DI) and one for muscle mass (MWI)—by dividing supraspinatus measurements by the sum of infraspinatus and teres minor measurements. When applying a similar index calculation to our data, we found that our measurements generally align with those of Bello‐Hellegouarch et al. (2013), suggesting consistency between the two datasets. Only the 3DI data of the single *Hylobates* specimen in each study did not fully correspond between the two studies. Bello‐Hellegouarch et al. (2013) concluded that a high 3DI did not necessarily indicate a high MWI, as exemplified by *Pan*, which exhibited a high 3DI but a low MWI. Therefore, the authors suggested that the muscle origin area of the dorsal rotator cuff is a weak indicator of muscle mass and, by extension, muscle volume.

In contrast, we argue that there is a correlation between muscle origin area and volume, and that this relationship can be particularly informative for comparative anatomical studies. There are several reasons why this relationship may have been overlooked in previous studies. First, we are convinced that the actual muscle attachment area, rather than the overall fossa size, is tightly correlated to muscle (mass and) volume, which is consistent with the findings of Larson and Stern (2013). Many earlier studies relied on linear measurements or landmark dimensions to approximate fossa size, which introduced significant variability and lacked a clear biological relationship with muscle volume. These measurements often included attachment areas of other muscles, further confounding the results. Recognizing the importance and difficulty of accurately determining muscle attachment sites on the scapula, we are publishing all muscle origin area meshes along with the 3D meshes of the scapulae in an online repository to guide future research efforts (van Beesel, Melillo & Vereecke, 2024).

Second, many previous studies have used aggregated datasets to compare fossa area and muscle mass or volume, with data obtained from different specimens (Larson & Stern, 2013). Importantly, in our study, we have correlated muscle volumes to origin areas of the same individual, using a consistent protocol. We found significant variation in origin area sizes and muscle volumes, as well as a hyperallometric correlation across genera. These findings suggest that it is not feasible to directly compare the relative origin area of one individual with the relative mass or volume of another, nor is it appropriate to use means across larger samples (as done in Figure 5a) since the relationship between origin size and muscle volume is not isometric.

Previous studies have often aimed to identify anatomical and functional differences between hominoid genera by comparing fossa sizes and their relationship to muscle volumes. However, given the small sample sizes (typically 1–4 specimens per genus) and the resulting high variability within these groups, as indicated by the large error bars in Figure 5a, this approach may have contributed to the inconsistencies observed in the findings. Instead, our study takes a broader approach by analysing hominoids as a single group, focusing on the commonalities across genera rather than emphasizing differences. Our results reveal a consistent pattern across hominoids, suggesting a genetic and/or developmental link between muscle attachment areas and their corresponding volumes.

Lastly, and perhaps most crucially, the specific muscles included in the analysis significantly impact the results and conclusions. Larson and Stern (2013) already noted that excluding the teres minor from comparisons greatly improved the accuracy of predictions. Our data corroborate this, showing substantial variability in teres minor origin areas and corresponding muscle volumes (see Figure 4a and the Data S1). Removing the teres minor (and major) from our analysis greatly enhanced the correlation, indicating that if a relationship exists between origin area and muscle volume for the teres minor, it likely follows a different slope or regression line than the other rotator cuff muscles.

What sets our study apart is the inclusion of the subscapularis muscle in the analysis. In Figure 5b, we calculated individual linear regressions between supraspinatus and infraspinatus muscle origin areas and volumes for each specimen in our dataset. The variation in regression slopes, particularly between *Pongo* and *Pan* (compare pink to red), was striking. These genera often presented conflicting results in previous studies, further complicating the interpretation of muscle origin and mass indices. However, when the subscapularis muscle was included in the analysis (Figure 5c), the slopes of the individual regressions converged, indicating a more consistent relationship. This suggests that while the supraspinatus and subscapularis muscle volumes are primarily constrained by bony structures—such as the extensions of their respective fossae and, in the case of the supraspinatus, the scapular spine—the infraspinatus muscle is likely more influenced by surrounding muscles. Specifically, the volumes of the teres major and teres minor, which extend directly adjacent to the infraspinatus across the axial border, may have a significant impact on its volume. Therefore, the relationship between infraspinatus origin area and volume may differ slightly from that of the supraspinatus and subscapularis, due to the greater influence of the surrounding muscles on the infraspinatus. In summary, by focusing on the actual attachment area rather than overall fossa size, comparing origin area and muscle volume within the same individuals, analysing across all hominoids, excluding the teres minor, and including the subscapularis muscle, we arrive at different conclusions than previous studies. Based on our findings, we can conclude that the origin area and volume of the supraspinatus, infraspinatus, and subscapularis muscles are indeed correlated.

### 4.4 Limitations of our study

One key limitation of this study is the small sample size, both in terms of evaluating the effectiveness of reconstructing muscle volumes from surface scans and in correlating muscle origin area with muscle volume. At the same time, the sample we present here is unique given that we include surface scans of muscles, CT scans of the scapula and humerus, and dissection data of nine hominoid primates. Given the difficulties in obtaining access to hominoid specimens, these data are incredibly valuable, and we are therefore sharing the raw data open access along with this paper.

Unfortunately, our sample was too small to investigate interspecific differences in the origin area to volume ratio or to examine effects of age or sex. However, the individual linear regressions shown in Figure 5c indicate that within‐species variation may be comparable to between‐species variation, suggesting a general pattern consistent across all hominoids. Nevertheless, we hope that future studies will build upon this dataset to further refine our understanding.

As discussed, the specific combination of muscles chosen for our reconstructions likely influenced the accuracy of the volume estimates. For instance, the teres major muscle reconstructions may have benefited from including a greater number of surrounding muscles. The relatively high error in the teres major volume estimates suggests that the method may struggle to accurately reconstruct muscles without clear skeletal boundaries. Whether incorporating more surrounding muscles or exploring alternative methods would improve these reconstructions will need to be addressed in future studies.

While our focus in this study was on the correlation between muscle origin area and muscle volume, which is crucial for reconstructing muscle masses in extinct species and determining muscle lines of action, we did not explore the correlation between muscle origin area and physiological cross‐sectional area (PCSA). A correlation with PCSA would be particularly valuable in paleontology, as PCSA is directly related to a muscle's force capacity. Interestingly, the relationship between muscle origin area and PCSA has been investigated across other vertebrates for different muscle groups, such as lower limb muscles and masticatory muscles, with varying degrees of success (Bates et al., 2021; Cuff et al., 2022; Toro‐Ibacache et al., 2015). Given the challenges we faced in accurately reconstructing muscle length—a necessary step for determining anatomical cross‐sectional area (Eigen & Nyakatura, 2021), which relates to PCSA—it is indeed likely that correlating muscle origin area with PCSA may be more complex. Nevertheless, this represents an intriguing area for future research, alongside investigating whether such correlations can be translated to other extant primates and whether similar relationships are present in other muscles.

## 5 CONCLUSION

This study demonstrates that, in hominoid primates, muscle volumes can be estimated from surface scans of dissected muscle layers, and that the muscle volume of the supraspinatus, infraspinatus, and subscapularis muscles can be estimated with reasonable accuracy from the origin area on the scapula. By refining previous methodologies—focusing on precise attachment areas, ensuring consistency within individual specimens, and including the subscapularis muscle in the analysis—we have revealed a strong correlation between muscle origin area and volume across hominoid species. This relationship holds significant potential for reconstructing muscle volumes in extant, and possibly also extinct, hominoids, providing a valuable tool for functional anatomical studies and paleontological research. Future work should explore whether similar correlations exist in other muscle groups and taxa, particularly those with large volumes and broad attachment sites.

## AUTHOR CONTRIBUTIONS

**Julia van Beesel:** Devised the study; evaluated the 3D datasets; led the data collection; wrote the Python scripts; evaluated the data; and wrote the manuscript. **Evie Vereecke and Stephanie Melillo:** Acquired funding for the data collection; acquired cadaver specimens for the data collection; reviewed and improved the data evaluation; provided comments on the manuscript; and approved final version of the manuscript.

## FUNDING INFORMATION

This project was funded by the Deutsche Forschungsgemeinschaft (DFG, German Research Foundation)—Project number 517064266 grant to JVB, by the Mercyhurst University Faculty Development Grant to SMM, by the Research Foundation Flanders (G076924N) to KU Leuven and by the former Department of Human Evolution of the Max Planck Institute for evolutionary Anthropology.

## CONFLICT OF INTEREST STATEMENT

The authors declare that they have no potential conflict of interest.

## Supporting information


Data S1.

## Data Availability

The data, accompanying python code as jupyter notebooks, and 3D meshes are available to view online (https://juliavanbeesel.github.io/ShoulderMuscleReconstructions/intro.html) and as download through the Zenodo repository: https://doi.org/10.5281/zenodo.13381978.
