# Supplementary material for: 3D reconstruction of shoulder muscles in hominoid primates: Correlating scapular attachment areas with muscle volume
Source: J Anat. 2025 Jan 4;246(5):709–23. doi: 10.1111/joa.14199 (PMC11996712; doi:10.1111/joa.14199)
Supplement: Supplementary file 1 — Data S1. [file JOA-246-709-s001.pdf]

Supplementary Information
3D Reconstruction of Shoulder Muscles in Hominoid Primates: Correlating Scapular Attachment Areas with Muscle Volume

Julia van Beesel[1,2,3], Stephanie Melillo[4] and Evie Vereecke[1]

[1]Department of Development and Regeneration, KU Leuven, Belgium
[2]Biomechanical Engineering, TU Delft, The Netherlands
[3]Department of Human Origins, Max Planck Institute for Evolutionary Anthropology, Germany
[4]Department of Applied Forensic Sciences, Mercyhurst University, PA, USA

1 Correlation results of teres major and minor

The correlation between the log-transformed origin area and the experimental muscle volume for the teres major and minor muscles was evaluated using Pearson's correlation coefficient (Figure 1). The analysis showed a moderate, positive correlation between the two variables, r=0.66, while the low p-value confirms that the correlation coefficient is significantly different from zero (p<0.01). The degrees of freedom for this test were df=15 (n=17).

These findings suggest that muscle origin area of the teres major and minor muscles is correlated to muscle volume. As the slope of the regression line equation is close to one (y = 1.08x + 2.00), this relationship appears to be isometric. However, the boundaries of the 95% confidence interval for the pearson correlation are quite large (CI: 0.26, 0.86), suggesting a high degree of uncertainty regarding the strength of the correlation. Therefore, the results should be treated with caution.

2 Tests for normality for correlation analyses

We performed Shapiro-Wilk tests before attempting the correlation analysis between the origin area and volume for the supraspinatus, infraspinatus and subscapularis muscles to test for normality of our dataset. The test results of both muscle origin area (Statistic: 0.99, p-value: 0.99) and muscle volume (Statistic: 0.98, p-value: 0.94) support the assumption for a

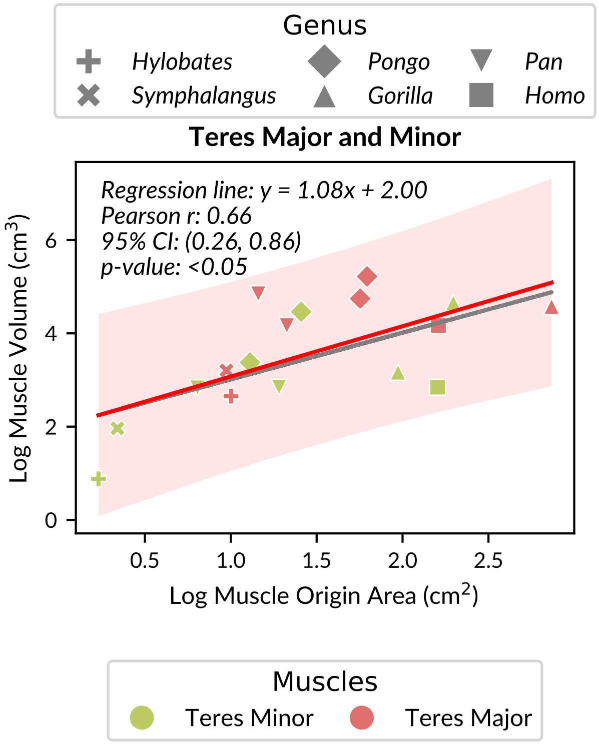

SI Figure 1: Pearson correlation of logarithmic transformed origin area and volume of teres major and minor muscles. The red line represents the best-fit linear regression line between muscle origin area and volume. The red shaded area around the red line indicates the prediction intervals. The grey line represents the prediction assuming isometric scaling with a slope of one.

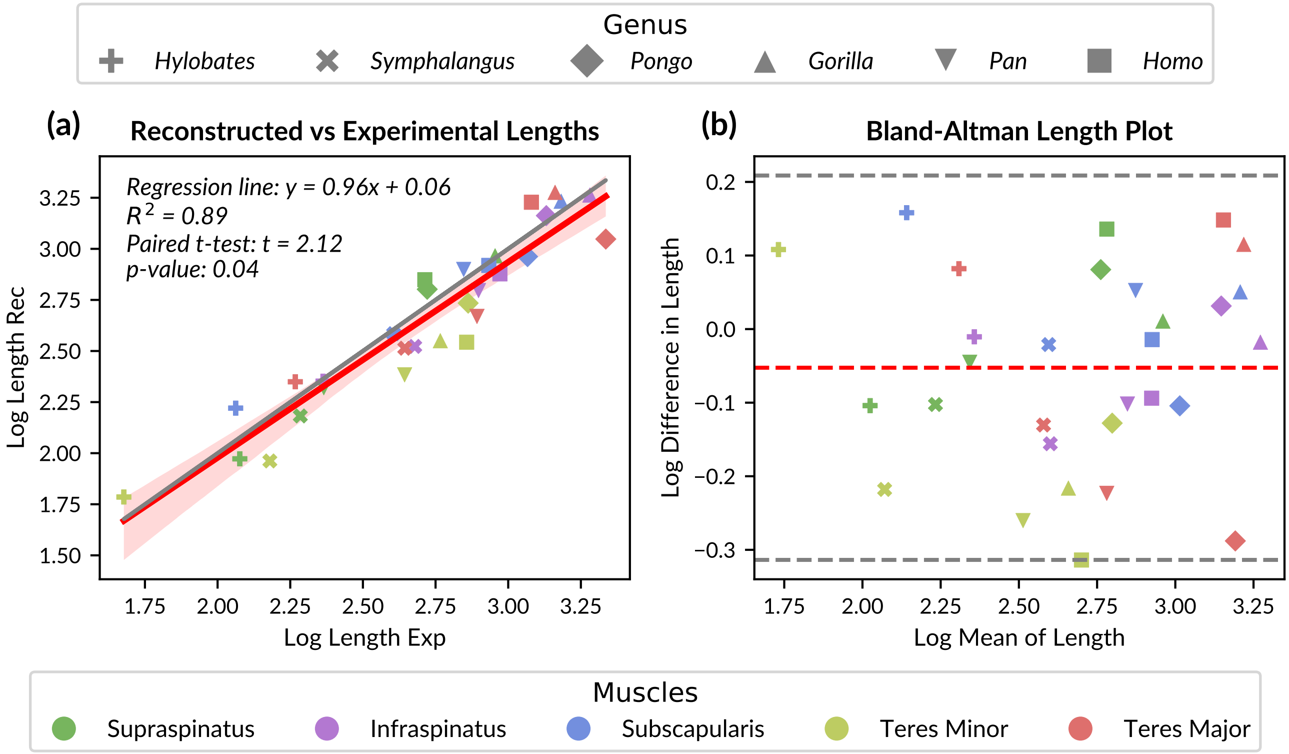

SI Figure 2: Accuracy of muscle length reconstructions. (a) Linear regression of reconstructed (rec) muscle lengths compared to experimental (exp) ones. The red line illustrates the optimal linear regression fit, surrounded by a red shaded area representing the 95% confidence interval of that regression. The grey line represents the identity line. (b) Bland-Altman plot displaying the differences between log-transformed reconstructed and experimental muscle lengths. The mean difference between the two measurements is shown by a red dashed line, while the grey dashed lines indicate the limits of agreement, which are determined as the mean difference ± 1.96 times the standard deviation of the differences.

normal distribution.

We further performed Shapiro-Wilk tests to test for normal distribution across the teres major and minor muscle origin areas and volumes. Similar as before, the test results of both muscle origin area (Statistic: 0.98, p-value: 0.92) and muscle volume (Statistic: 0.94, p-value: 0.29) support the assumption for a normal distribution.

3 Accuracy of estimating Muscle Length from 3D reconstructions

The paired t-test suggests a significant difference between the log-transformed experimental lengths and log-transformed polygonal lengths (t-statistic: 2.12, p-value: 0.04). The mean absolute error (MAE: 0.12) and root mean squared error (RMSE: 0.14) on the log-transformed scale are relative high. The coefficient of determination is R^2: 0.869 (see (Figure 2(a)). Given the low p-value, we reject the null hypothesis (H_0_1), suggesting that there is a significant average difference between the methods.

The mean difference between the reconstructed lengths and the experimental lengths is negative (-0.05), indicating that the reconstructed lengths generally underestimated the experimental lengths (Figure 2(b)). This is further indicated by the regression line lying below the identity line (Figure 2(a)). The data points are equally distributed across the Bland-Altman plot, indicating a systematic error when measuring muscle lengths from the reconstructed polygons. Notably, all but one teres minor reconstructed muscle lengths underestimate the experimental muscle lengths.
